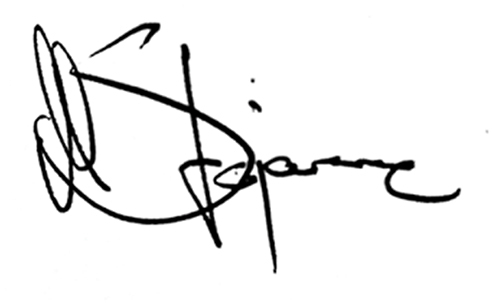# Welcome to Interventional Pain Medicine

**DOI:** 10.1016/j.inpm.2022.100081

**Published:** 2022-03-08

**Authors:** Milan P. Stojanovic

**Affiliations:** Interventional Pain Medicine

It is my great honor and privilege to introduce the inaugural issue of the journal *Interventional Pain Medicine*, the Official Journal of the Spine Intervention Society (SIS).

Over the last 50 years, since the Melzak and Wall theory changed our understanding of pain, we have witnessed a rapid expansion of a new subspecialty field – pain medicine. Previous “trial and error” methods gave way to evidence-based medicine, neuroscience delivered new medications and neuromodulation techniques, psychology provided insights on pain coping skills, and rehabilitation enhanced functional improvement techniques. Detailed anatomical dissections and imaging advancements helped us develop precise interventions to diagnose and treat pain. With the expansion of fellowship programs and increased diversity of trainees and training backgrounds, the multidisciplinary approach to diagnosis and treatment of complex pain conditions became the standard of care.

Starting a new journal, *Interventional Pain Medicine*, with a narrow focus may at first seem like a departure from traditional multidisciplinary efforts. To be clear, I am a strong supporter of this tradition. However, expansion and advancement in a field often leads to greater specialization. As our specialty has grown, the field of “interventional pain medicine” has experienced commensurately greater scientific innovation, from improvements in the safety and effectiveness of existing therapies, to novel therapeutics, and the creation of minimally invasive procedures that bridge the gap between surgical and non-surgical treatments. Dissemination of standardized procedural techniques through accredited training programs and dedicated societal efforts have led to exponential growth in the use diagnostic and therapeutic interventions, which has not always resulted in better individual and societal outcomes. Interventional pain medicine in many ways is the beating heart of the specialty. To accompany the rapid growth in the numbers of procedures and providers, a dedicated platform is needed to assess, validate and promote scientific advancements in the field, to assure an evidence-based approach to patient care, and to serve as a communication hub. With these ideas in mind, the journal *Interventional Pain Medicine* was born.

The goal of *Interventional Pain Medicine* is to provide an intellectual home for physicians and researchers across specialties, and to provide answers to scientific and clinical questions within this rapidly evolving field. The Journal is fully vested in the diversity of our specialty, journal contributors and editorial board in all shapes and forms. We are similarly dedicated to helping young and novice researchers improve scientific standards by offering a comprehensive review process with attention to details. Although the Journal's main focus is original research related to mechanisms, techniques and clinical outcomes of new and existing interventional procedures for all types of pain conditions (e.g., spine, musculoskeletal, headaches, cancer pain, neuromodulation, minimally invasive surgery and regenerative medicine), the Journal also welcomes studies on basic science, anatomy, creative concepts and clinical advancements that underpin the field of interventional pain medicine.

One of the Journal's goals is to improve traditional publishing with the new modes of communication available through modern technology. For example, we will accept video presentations conducive to viewing on computers and mobile devices either as a supplement to an original manuscript or a stand-alone presentation in our “multimedia section.” With the rapid decline of print journals, shifting to electronic devices expands the Journal's reach and makes manuscripts more accessible to readers around the world. Considering these points, *Interventional Pain Medicine* decided to launch as an open access journal to make manuscripts available “free of charge”, thereby increasing our global footprint. Although our goal is to waive or reduce the Article Publishing Charge (APC) for select high-quality scientific papers in the future, during the Journal's launch APCs are waived for all accepted manuscripts.

I enthusiastically believe that *Interventional Pain Medicine* will fulfil its mission and become one of the leading scientific journals in the field. I firmly believe that the Journal will develop into the platform of choice for interventional pain physicians and researchers to present and promote their work and become a nexus learning for interventional pain physicians at all levels of experience. I am further convinced that it will soon be recognized for its impeccable scientific standards, diversity, and dedication to innovation – all qualities that reflect its parent organization, the Spine Intervention Society.

Sincerely,

**Figure:**